# Harnessing Light Quality for Potato Production: Red and Blue Light as Key Regulators of Growth and Yield

**DOI:** 10.3390/plants14071039

**Published:** 2025-03-27

**Authors:** Rong Guo, Yanjun Jin, Juan Liu, Hongyu Yang, Lixiang Cheng, Bin Yu

**Affiliations:** 1 State Key Laboratory of Aridland Crop Science, Gansu Agricultural University, Lanzhou 730070, China; guor112930@163.com (R.G.);; 2 College Agronomy, Gansu Agricultural University, Lanzhou 730070, China; 3 College of Water Conservancy and Hydropower Engineering, Gansu Agricultural University, Lanzhou 730070, China; 4 College of Horticulture, Gansu Agricultural University, Lanzhou 730070, China

**Keywords:** potato, light quality, plant physiology, tuber yield, agricultural light environment

## Abstract

This study aimed to investigate the effects of different light qualities on the morphological development, photosynthetic characteristics, stomatal structure, and yield of potato, providing theoretical and practical guidance for optimizing light environments in controlled agricultural systems and enhancing the efficient production of potato microtubers. Six light qualities—white, red, blue, green, far-red, and ultraviolet—were applied to systematically evaluate their effects. The results showed that light quality significantly influenced plant morphological traits and physiological metabolism. Red and blue light demonstrated the most pronounced promotive effects. Under red light, plant height and stem diameter increased by 57.47% and 31.10%, respectively, compared to white light, while single tuber weight increased by 20.09%, despite a 14.96% reduction in tuber number per plant. Blue light significantly enhanced chlorophyll content (by 20.35%) and stomatal density (adaxial stomata increased by 28.85%), leading to a 38.98% increase in tuber number, a 51.79% increase in single tuber weight, and a remarkable 110.37% improvement in total yield per plant, compared to white light. In contrast, green light moderately promoted photosynthesis in lower leaves, but reduced the total yield by 39.90%. Far-red (740 nm) and ultraviolet light (390 nm) severely inhibited plant growth and failed to induce tuber formation. Correlation analysis revealed a highly significant positive relationship between chlorophyll content, net photosynthetic rate, stomatal density, and yield per plant (r = 0.96, *p* < 0.01). This study systematically evaluated the independent effects of single light quality on potato growth and production for the first time, clarifying the regulatory advantages of red and blue light, and providing important theoretical insights for optimizing the light environment with red and blue light to improve potato microtuber yield. Furthermore, this study provides critical data to support future research on the dynamic optimization of light quality ratio.

## 1. Introduction

Light quality, defined by the range of wavelengths, not only influences photosynthesis, the process by which plants convert light energy into organic compounds, but also serves as an important growth and developmental signal. It regulates various physiological processes, such as morphological development, photoperiod responses, and secondary metabolism, through photoreceptors, making it a critical environmental factor in plant growth and development. Solar light falling on plants comprises light of different spectral regions, including ultraviolet B (280–320 nm) and A (320–400 nm), blue (400–500 nm), green (500–570 nm), yellow (570–590 nm), orange (590–620 nm), red (620–700 nm), and far-red light (700–800 nm).

Photosynthesis involves the conversion of carbon dioxide and water into organic matter using light energy. In this process, Photosystem I and Photosystem II absorb light of different wavelengths. Blue and red light are the main components of photosynthetically active radiation and significantly contribute to photosynthesis. Studies show that red light enhances the electron transfer efficiency of Photosystem II, increasing its activity and promoting ATP and NADPH production, which, in turn, enhances carbon assimilation and photosynthetic efficiency [1]. Blue light promotes chlorophyll synthesis, improving light absorption and energy conversion efficiency. It also activates blue light receptors in guard cells, enhancing cell expansion, accelerating stomatal opening, and increasing stomatal conductance, thereby improving photosynthesis [2]. Although ultraviolet, green, and far-red light contribute less to photosynthesis, they have specific roles in the regulation of plant photosynthetic physiology. Ultraviolet light is classified into UV-A (320–400 nm), UV-B (280–320 nm), and UV-C (100–280 nm), with UV-A and UV-B being the most common types in the plant’s natural environment. UV-C is absorbed by the atmosphere, and typically does not affect plants directly. UV-A penetrates the plant leaf epidermis, activating chlorophyll and carotenoids. UV-B (280–320 nm) exerts a dose-dependent dual effect on plants: at low doses, it acts as a signaling molecule to activate protective mechanisms, whereas at high doses, it induces stress-related damage [3]. The only known UV-B photoreceptor in plants to date is the UVR8 (UV RESISTANCE LOCUS 8) protein. It induces photomorphogenetic effects and works at low UV-B doses. The plant response to UV-B is influenced by the wavelength, intensity, and duration of radiation, and UV-B is detected by photoreceptors such as phytochromes and cryptochrome. At low doses, the protective response is activated via signaling pathways mediated by these photoreceptors, promoting photomorphogenesis, inducing flavonoid synthesis, and enhancing the antioxidant system [4,5]. In contrast, high-dose UV-B induces DNA damage and ROS production, and inhibits photosynthesis [6]. Green light was once thought to have a minimal effect on plant growth, as most green light is reflected by the leaves. However, this perception has limitations that need to be corrected. Recent studies have shown that green light penetrates deeply into the lower tiers of the plant, and contributes to the photosynthesis of shade leaves. In a study on green light treatment in pepper cultivation, the results showed that the light intensity in the deeper layers of the canopy increased by 43% to 158% compared to in conventional conditions [7]. In addition, green light can affect stomatal movement through the synergistic action of photoreceptors and other photosensitive substances. Green light indirectly influences stomatal movement via cryptochrome and phototropin, although the effect is relatively small. In most cases, green light works synergistically with red and blue light to regulate stomatal opening and closing [8,9]. Far-red light regulates plant morphology and photoperiod responses by interacting with phytochrome receptors to trigger shade avoidance responses, such as stem elongation and changes in leaf orientation, thereby affecting resource allocation and crop yield [10]. The optimal combination of far-red light and red light can enhance photosynthetic efficiency, especially under low-light conditions [11]. Supplementation with appropriate far-red light can further improve light energy utilization [12]. Conversely, excessive far-red light induces exaggerated shade avoidance, resulting in over-elongated stems, reduced mechanical strength, increased lodging risk [13], and reduced chlorophyll content, thus weakening photosynthetic capacity [14].

Plant morphology is regulated by different light qualities, especially during seedling and early growth stages, with light quality significantly shaping morphological traits. Studies show that blue light, by activating cryptochromes and phototropins, regulates auxin distribution, suppressing stem elongation and promoting even leaf distribution [15]. Blue light also promotes root development and increases root hair density, aiding in water and nutrient absorption [16]. Red light facilitates stem elongation, flowering, and fruiting. Research indicates that red light enhances the synthesis and transport of auxins, accelerating vertical stem growth and influencing photoreceptor activity to affect photoperiod responses and the biological clock [17]. Far-red light induces the shade avoidance response, promoting stem elongation to allow the plant to capture more light, but also reducing leaf area and inhibiting root development [18]. Far-red light activates the far-red absorbing form (Pfr) of photoreceptors, regulates the photoperiod perception system, and affects flowering and growth rate [19]. Although green light is often neglected due to its reflection by leaves, some studies suggest that increasing green light reduces lateral branching, suppresses bud growth, and promotes leaf expansion [20].

With the advancement of modern agriculture, artificial lighting technology plays an increasingly significant role in plant production, particularly in facility agriculture, such as in greenhouse cultivation, plant factories, and vertical farms. Light-emitting diodes (LEDs), known for their energy efficiency and controllable light quality and intensity, have been widely used in facility agriculture. Research indicates that red light accelerates the accumulation of capsaicin in chili peppers and promotes color change in pepper fruits [21]. Blue light treatment enhances cucumber seedling leaf development, increasing stomatal density, and thereby improving net photosynthetic rate, promoting growth, and improving cucumber yield and quality [22]. Green light treatment can penetrate cucumber leaves, acting on the lower leaves, promoting their photosynthesis, and enhancing dry matter accumulation, thus improving cucumber yield and quality [23]. Adding far-red light to white light to adjust the R/FR ratio improves the plant height and leaf area of tomato plants, as well as their soluble sugar and vitamin C content, thus increasing yield [24].

Potato (*Solanum tuberosum* L.) is the fourth largest food crop in the world, and plays an important role in ensuring global food security [25]. The production and application of virus-free seed potatoes are critical for increasing potato yield. LED-based light factory production of virus-free seed potatoes is an important direction for efficient potato production. This experiment uses the widely cultivated potato variety ‘Atlantic’ as the study material, and explores the effects of six light quality treatments (white, far-red, red, green, blue, and ultraviolet) on plant morphology, photosynthetic properties, stomatal characteristics, and yield. The aim is to understand the role of different light qualities in potato growth, and to provide a theoretical basis for modern agricultural light management and light environment optimization in seed potato production.

## 2. Materials and Methods

### 2.1. Experimental Design

The potato variety ‘Atlantic’ was used as the material in this experiment. The tissue culture seedlings were provided by the State Key Laboratory of Aridland Crop Science. The experiment was carried out in the greenhouse of Gansu Agricultural University. A pot experiment was conducted in a controlled environment with the same conditions in an artificial climate chamber. Before transplanting, tissue culture seedlings were propagated and acclimatized for 48 h when they reached 21 days of growth. Healthy, sterile seedlings with well-expanded leaves were selected and planted in pots containing 200 g of vermiculite (diameter = 12 cm, depth = 10 cm). After 14 days of acclimatization, 20 pots with uniform plant growth were selected for different light quality treatments in the artificial climate chamber; there were 2 plants per pot, and 40 plants in total. After 14 days of acclimatization, the 3-leaf stage was reached, and 20 pots with uniform growth were selected and treated with different light qualities in the artificial climate chamber for 60 days. The substrate in the pots was vermiculite, and the nutrient solution formulation was MS formulation, which was irrigated every 15 d during the experimental period at a concentration of 2:1, with a temperature of 25 ± 3 °C and an air humidity of 60% during growth.

### 2.2. Light Quality Settings

The artificial climate chamber (dimensions: 0.6 m × 0.6 m × 0.5 m) was equipped with LED light panels (size: 500 mm × 500 mm × 8 mm) that allowed for controlled light intensities (0–5000 LX). The plants were subjected to light with different spectral compositions in individual boxes of the climatic chamber: white (maximum of 400–760 nm, 21–150 nm FWHM), far-red (maximum of 740 nm, 100 nm FWHM), red (maximum of 660 nm, 24 nm FWHM), green (maximum of 520 nm, 30 nm FWHM), blue (maximum of 450 nm, 26 nm FWHM), and ultraviolet light (maximum of 390 nm, 40 nm FWHM) LEDs (LH-T8120L-1200, Aislang Lighting Limited Company, Jiangmen, China) (Table 1). The ultraviolet light employed was UV-A. The light intensity was 300 μmol (photon)/m^2^s. A single light source was applied to each treatment throughout the entire growth cycle of the plants. The photoperiod was set to 12 h (07:00–19:00), with the remaining time in darkness.

Data collection: Plant height, stem diameter, stomatal characteristics, and photosynthetic parameters were measured one day before light treatment. After light treatment, these indexes were measured every 5 days, for a total of 7 times. Three replicates were performed for each treatment, and the third leaf of each plant was selected for measurement. Pn, Chl, and Tr values were averaged over seven measurements. At the seventh sampling, root length and root surface area were also measured. Five pots from each treatment were selected for yield assessment after the plants reached maturity.

### 2.3. Measurement of Morphological and Yield Trait Profiling

Above-ground traits, below-ground traits, and yield-related parameters were evaluated to understand the growth and productivity of potato plants under different treatments. Plant height was measured using a ruler as the vertical distance from the stem base to the apex. Stem diameter was measured at the third internode using a vernier caliper. For below-ground traits, root length and root surface area were analyzed on day 35 post treatment using a root scanner (EPSON, J241A, JPN). After full maturity, yield-related parameters, including the number of tubers per plant and total yield per plant, were recorded using standard harvesting and calculation methods.

### 2.4. Measurement of Photosynthetic Parameters

The photosynthetic rate was measured between 9:00 and 11:00 a.m., using a portable photosynthesis system (LI-6400XT, LI-COR, Lincoln, NE, USA) on potato plants in the growth chamber. For each treatment, three plants were measured, selecting the top leaflets of the third leaf. Three points were selected on each leaflet, avoiding the veins, and readings were taken at each point.

### 2.5. Measurement of Stomatal Parameters

Abaxial Leaf Stomatal Imprints: A thin layer of transparent nail polish was applied evenly on the abaxial leaf surface. The nail polish was allowed to dry for 25 min, and then was removed with tweezers. The imprint with epidermal cell patterns was cut and placed on a glass slide with a drop of distilled water for observation.

Adaxial Leaf Stomatal Imprints: The main vein of the leaf was removed, and the leaf was laid down with the abaxial surface facing up. A 5 cm piece of adhesive tape was applied from the leaf tip to the base, pressing the tape to ensure good adhesion. After slowly separating the tape, the abaxial epidermis adhered to the tape. The required section was cut and placed on a glass slide with a drop of distilled water for observation.

Stomatal Density Observation: Stomatal density was observed using an inverted fluorescence microscope (RVL-100-G, ECHO, San Diego, CA, USA). Five fields of view (20 × 10) were selected per slide for imaging, and stomata were counted across the entire field of view.

$$Stomatal\;density\;(n/{mm}^{2})=\frac{The\;number\;of\;stomata\;(N)(n)}{The\;measuring\;area\;(M)({mm}^{2})}$$

Stomatal Size Measurement: Stomatal size was measured using an inverted fluorescence microscope. For each slide, five different fields of view (20 × 10) were photographed, and 20 randomly selected stomata were measured for length and width.

### 2.6. Measurement of Chlorophyll Content

A 0.5 g leaf sample was weighed, chopped, and placed into a test tube. Then, 15 mL of anhydrous ethanol was added, and the mixture was shaken thoroughly to ensure the leaf fragments were completely submerged in the ethanol. The test tube was then heated in a water bath at 60 °C for 2 h, until the leaves turned completely white, after which the mixture was allowed to cool naturally. The chlorophyll content was measured using a spectrophotometer, with anhydrous ethanol as a blank control. Absorbance was measured at wavelengths of 645 nm and 663 nm.

$$\mathrm{Chl}a=12.7D_{663}-2.69D_{645}$$

$$Chlb=22.9D_{645}-4.68D_{663}$$

$$\mathrm{Chl}a+\mathrm{Chl}b=20.21D_{645}+8.02D_{663}$$

Chl*a* represents the content of chlorophyll a; Chl*b* represents the content of chlorophyll b; Chl*a* + Chl*b* represents the total chlorophyll content.

### 2.7. Leaf Anatomical Structure Observation

The methods for assessing the anatomical characteristics of leaves were developed with reference to Chen et al. [26]. For leaf anatomy observation, the third unfolded leaf of the plant was taken, and each treatment was replicated three times. Fixation and Dehydration: Leaf samples (0.5 cm × 0.5 cm) were immersed in 10 mL Formalin-Acetic Acid-Alcohol Fixative (FAA) fixative (90 mL 70% ethanol + 5 mL glacial acetic acid + 5 mL formaldehyde) at room temperature for 24 h. Fixed samples were dehydrated sequentially in ethanol solutions of increasing concentrations (70% → 80% → 90% → 95% → 100%). The dehydrated material was placed in pure xylene until no white cloudy precipitate was visible, then was processed further.

Clearing and Wax Infusion: Dehydrated leaf samples were immersed in a solution of anhydrous ethanol and xylene (V:V = 1:1) for 2 h, followed by three rounds of clearing in pure xylene. The samples were then immersed in a 55 °C solution of paraffin and xylene (V:V = 1:1) for 8 h. Subsequently, the samples were transferred to 65 °C conditions and immersed in 100% paraffin for 16 h, with the paraffin replaced every 2 h.

Embedding and Sectioning: Paraffin blocks were cut into small pieces, melted in a beaker, and samples were embedded at 65 °C using an embedding machine. Embedded samples were sectioned with a rotary microtome, and the sections were floated on water at 37 °C for 72 h.

Dewaxing and Staining: Sections were dewaxed using the following series of solutions: 100% xylene → anhydrous ethanol and xylene (V: V = 1:1) → 100% ethanol → 90% ethanol → 80% ethanol → 70% ethanol. Staining was performed using safranin and fast green solutions in the following sequence: safranin → 80% ethanol → 90% ethanol → fast green → 95% ethanol (twice) → anhydrous ethanol. Sections were mounted with diluted resin adhesive, covered with cover slips, labeled, and observed using an inverted fluorescence microscope (RVL-100-G, ECHO, San Diego, CA, USA). Five different fields of view (20 × 10) were photographed per slide.

### 2.8. Data Analysis

Data organization was performed using Excel 2019, charting was performed using Origin 2024, and statistical analysis of data was performed using SPSS 23.0, with one-way analysis of variance (ANOVA) for each indicator.

## 3. Results

### 3.1. Effects of Light Quality on Potato Plant Growth

**Figure 1:**
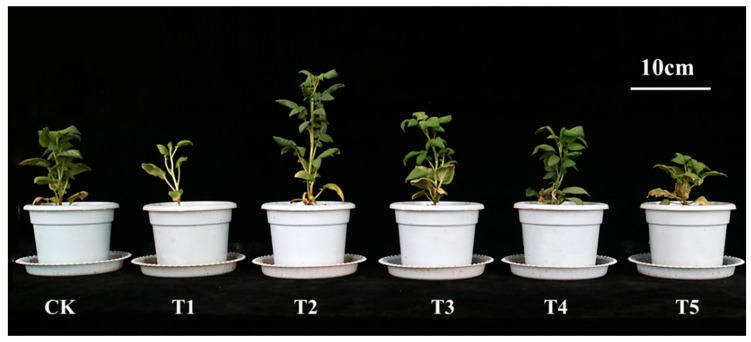
The growth morphology of potato plants after 20 days under different light quality treatments. Note: CK: white light treatment; T1: far-red light treatment; T2: red light treatment; T3: green light treatment; T4: blue light treatment; T5: ultraviolet light treatment.

**Figure 2:**
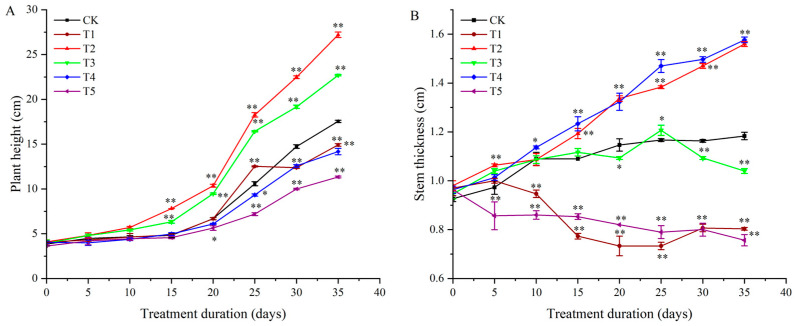
The effects of different light qualities on the stem growth of potato plants. (**A**): plant height of different light qualities of potato; (**B**): stem thickness of different light qualities of potato.Note: In the figure, “*” represents significant differences from the CK at the same time point (*p* < 0.05), and “**” indicates extremely significant differences (*p* < 0.01). CK: white light treatment; T1: far-red light treatment; T2: red light treatment; T3: green light treatment; T4: blue light treatment; T5: ultraviolet light treatment.

Red and green light significantly increased plant height compared to white light (Figure 1 and Figure 2A). Under red light, plant height increased by 60.42%, 54.41%, 70.75%, 51.35%, and 57.47% on days 15, 20, 25, 30, and 35, respectively. Green light increased plant height by 31.25%, 38.24%, 53.77%, 30.41%, and 29.89% over the same period. Blue, far-red, and ultraviolet light inhibited plant height. Blue light reduced plant height by 11.77%, 12.26%, 14.87%, and 18.39% from day 20 to day 35. Far-red light caused decreases of 14.87% and 14.37% on days 30 and 35. Ultraviolet light reduced plant height by 19.11%, 31.13%, 32.43%, and 33.91% from day 20 to day 35 (Figure 2A).

Red and blue light promoted stem thickness, while far-red and ultraviolet light inhibited it. Red light increased stem thickness by 17.70%, 19.13%, 26.50%, and 31.10% from day 20 to day 35. Blue light increased stem thickness by 13.89%, 18.58%, 26.09%, 28.21%, and 31.09% from day 15 to day 35. Far-red light reduced stem thickness by 26.85%, 36.28%, 35.65%, 31.62%, and 31.93% from day 15 to day 35. Ultraviolet light decreased stem thickness by 21.30%, 27.43%, 30.44%, 31.62%, and 36.13% over the same period (Figure 2B).

### 3.2. Effects of Light Quality on Anatomical Structure of Potato Leaves

**Figure 3:**
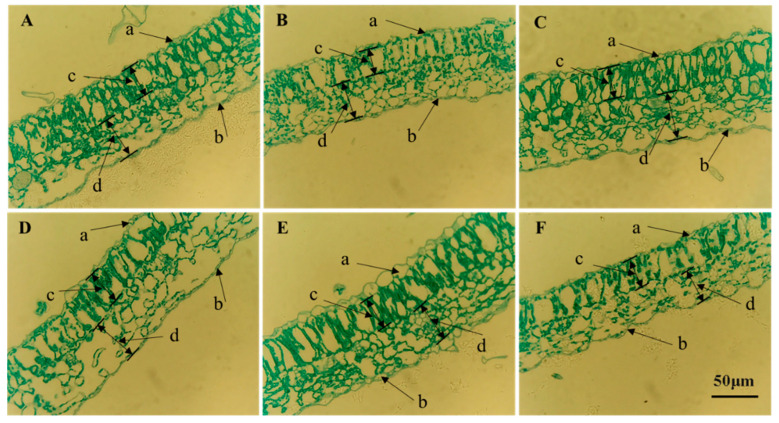
The effects of different light qualities on the anatomical structure of potato plant leaves. Note: (**A**): white light treatment; (**B**): far-red light treatment; (**C**): red light treatment; (**D**): green light treatment; (**E**): blue light treatment; (**F**): ultraviolet light treatment; a: upper epidermis; b: lower epidermis; c: fence tissue; d: sponge tissue.

The anatomical structures of potato leaves showed significant differences under various light quality treatments (Figure 3). Compared to white light, far-red and ultraviolet light treatments resulted in larger intercellular spaces in the leaves, along with reduced thickness of the upper and lower epidermis, palisade tissue, and spongy tissue. Additionally, the palisade and spongy cells were loosely arranged. Under red light treatment, leaf thickness increased due to an increase in spongy tissue thickness, with tightly arranged palisade cells. Green light treatment decreased the thickness of the palisade tissue, but increased the thickness of the spongy tissue, with loosely arranged palisade and spongy cells. Blue light treatment increased leaf thickness by enhancing the thickness of the upper and lower epidermis, with tightly arranged palisade and spongy cells.

### 3.3. Effects of Light Quality on Potato Root Growth

**Figure 4:**
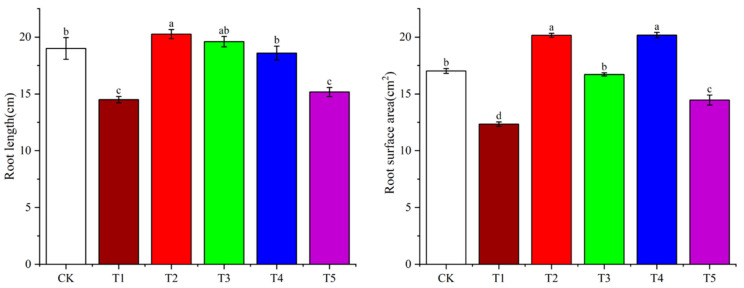
The effects of different light qualities on the growth of potato roots. Note: CK: white light treatment; T1: far-red light treatment; T2: red light treatment; T3: green light treatment; T4: blue light treatment; T5: ultraviolet light treatment. The bar chart data are represented as the mean ± standard error. Different lowercase letters indicate significant differences at *p* < 0.05.

**Figure 5:**
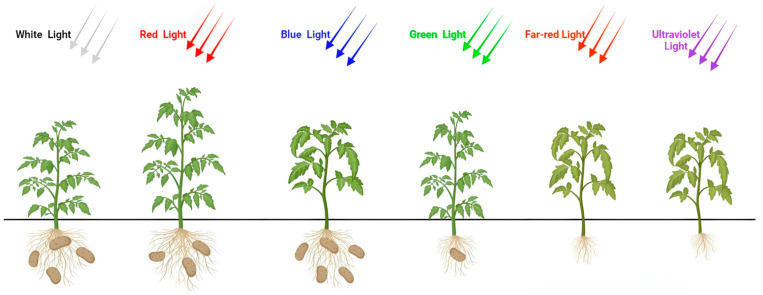
The effects of different light qualities on the growth of potato plants.

Different light quality treatments had significant effects on potato root length and root surface area (Figure 4 and Figure 5). Far-red light treatment inhibited both root length and root surface area in the Atlantic cultivar, with root length was significantly reduced by 23.20%, and root surface area by 27.64%, compared to white light. Under red light treatment, root length showed a significant change compared to white light, and root surface area significantly increased by 18.82%. Green light treatment had no significant effect on either root length or root surface area. Blue light treatment did not affect root length, but significantly increased root surface area by 18.24%. Ultraviolet light treatment suppressed root growth, with root length and root surface area reduced by 20.00% and 14.71%, respectively, compared to white light.

### 3.4. Effects of Light Quality on Photosynthetic Characteristics of Potato Leaves

Light quality significantly affected the chlorophyll content, net photosynthetic rate, and transpiration rate in potato leaves. The chlorophyll content followed the following order: blue light > green light > red light > white light > ultraviolet light > far-red light. Blue light increased the chlorophyll content by 20.35%, while ultraviolet and far-red light reduced it by 20.93% and 23.84%, respectively. No significant changes were observed under red or green light (Table 2).

The net photosynthetic rate decreased under some treatments, with reductions of 10.79%, 7.52%, 8.21%, 2.18%, for far-red, green, ultraviolet, and red, respectively. The transpiration rate varied, with blue light significantly increasing the rate by 21.60%, and far-red light significantly reducing the rate by 40.74%. Red, green, and ultraviolet light showed no significant impact on transpiration (Table 2).

### 3.5. Effects of Light Quality on Stomatal Characteristics of Potato Leaves

**Figure 6:**
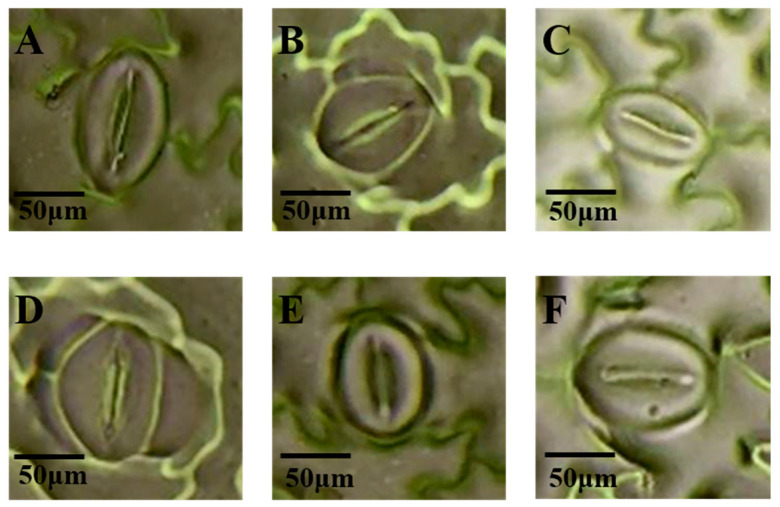
The effects of different light qualities on the stomatal size on the adaxial surface of potato leaves. Note: (**A**): white light treatment; (**B**): far-red light treatment; (**C**): red light treatment; (**D**): green light treatment; (**E**): blue light treatment; (**F**): ultraviolet light treatment.

Different light qualities influenced the stomatal size in potato leaves (Figure 6). On the adaxial surface, green light increased the stomatal width by 20.74% compared to white light, with no effect on stomatal length. Blue light increased the stomatal length and width by 16.56% and 16.66%, respectively, while ultraviolet light increased the stomatal length by 16.59%, without affecting the stomatal width. All treatments had no significant effects on the stomatal size on the adaxial surface (Table 3).

On the abaxial surface, the stomatal length followed the following order: blue light > far-red light > white light > ultraviolet light > red light > green light. Blue light significantly increased the stomatal length and width by 27.96% and 29.54%, respectively, compared to white light. Other treatments did not significantly affect the stomatal size on the abaxial surface (Table 3).

### 3.6. Effects of Light Quality on Stomatal Density of Potato Leaves

**Figure 7:**
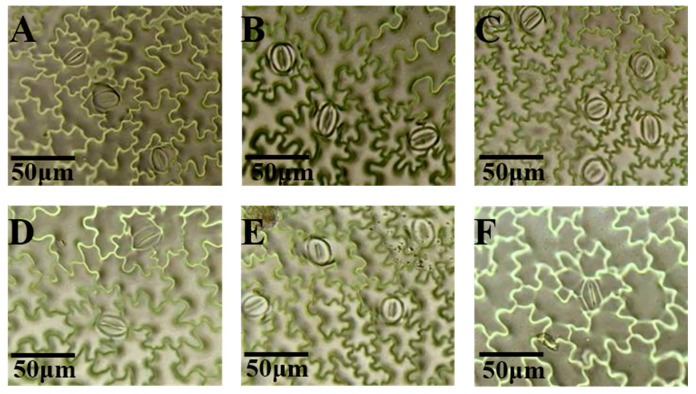
The effects of different light qualities on the stomatal density on the adaxial surface of potato leaves. Note: (**A**): white light treatment; (**B**): far-red light treatment; (**C**): red light treatment; (**D**): green light treatment; (**E**): blue light treatment; (**F**): ultraviolet light treatment.

**Figure 8:**
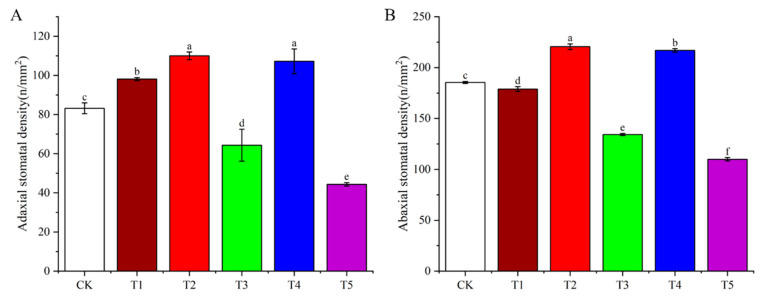
The effects of different light qualities on the stomatal density of potato leaves. (**A**): Adaxial stomatal density of potao leaves under different light qualities. (**B**): Abaxial stomatal density of potao leaves under different light qualities. Note: CK: white light treatment; T1: far-red light treatment; T2: red light treatment; T3: green light treatment; T4: blue light treatment; T5: ultraviolet light treatment. The data in the table are displayed as the average ± standard deviation; different lowercase letters indicate significant differences at *p* < 0.05.

The stomatal density on the adaxial surface of Atlantic potato leaves was significantly influenced by light quality (Figure 7). The far-red, red, and blue light treatments increased the stomatal density by 17.98%, 32.20%, and 28.85%, respectively, compared to white light. In contrast, green and ultraviolet light reduced the stomatal density by 22.64% and 46.61%, respectively (Figure 8A).

**Figure 9:**
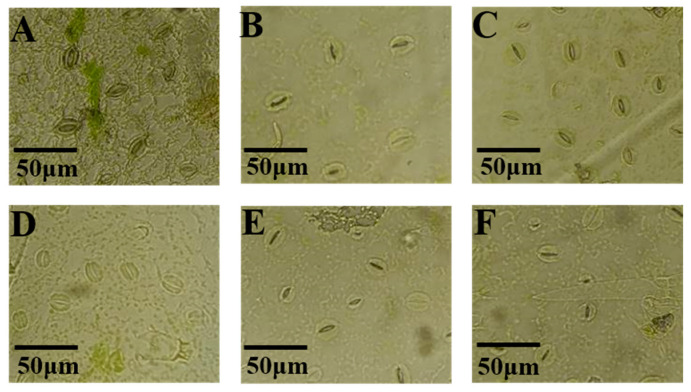
The effects of different light qualities on the stomatal density on the abaxial surface of potato leaves. Note: (**A**): white light treatment; (**B**): far-red light treatment; (**C**): red light treatment; (**D**): green light treatment; (**E**): blue light treatment; (**F**): ultraviolet light treatment.

On the abaxial surface, similar trends were observed (Figure 9). The red and blue light treatments increased the stomatal density by 18.92% and 16.88%, respectively, while green, far-red, and ultraviolet light decreased it by 27.01%, 3.54%, and 40.45%, respectively (Figure 8B).

### 3.7. Effects of Light Quality on Tuber Yield Traits

Different light qualities significantly affected yield traits in Atlantic potatoes (Table 4). Far-red and ultraviolet light treatments inhibited tuber formation, resulting in no tuber production. Under red light, the number of tubers per plant decreased by 14.96%, compared to under white light. Green light reduced the total yield by 39.90%, the number of tubers by 16.54%, and the average tuber weight by 28.57%. Blue light significantly enhanced the tuber yield, increasing the number of tubers by 38.98%, the average tuber weight by 51.79%, and the total yield by 110.37%, compared to white light.

### 3.8. Correlations Between Photosynthetic Characteristics and Yield Traits Under Different Light Qualities

**Figure 10:**
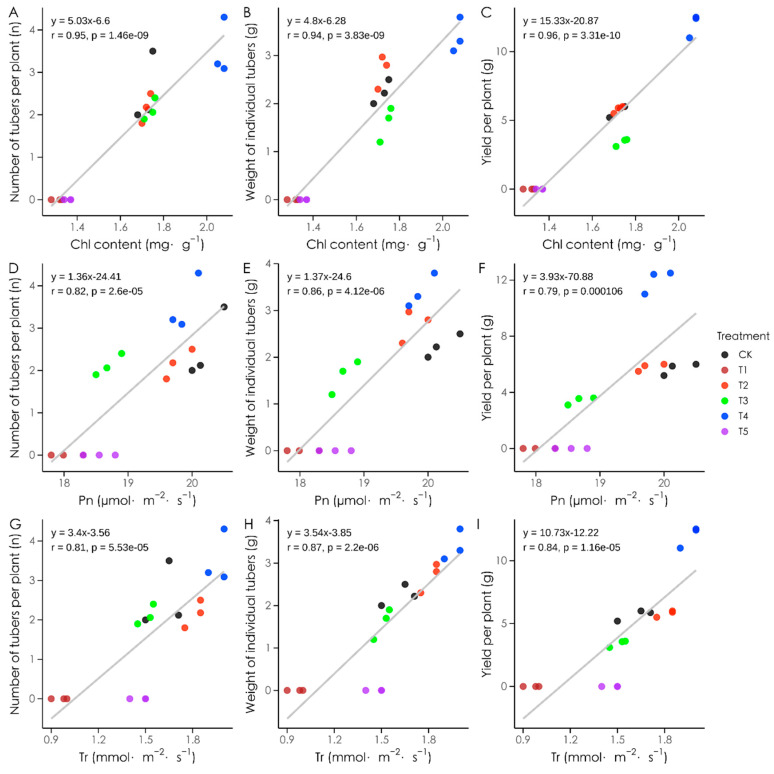
Correlation analysis of photosynthetic characteristics and yield traits of potato plants under different light qualities. Note: (**A**): correlation analysis between total chlorophyll content and number of potato tubers per plant; (**B**): correlation analysis between total chlorophyll content and weight of individual tubers; (**C**): correlation analysis between total chlorophyll content and yield per plant; (**D**): correlation analysis between net photosynthetic rate and number of potato tubers per plant; (**E**): correlation analysis between net photosynthetic rate and weight of individual tubers; (**F**): correlation analysis between net photosynthetic rate and yield per plant; (**G**): correlation analysis between transpiration rate and number of potato tubers per plant; (**H**): correlation analysis between transpiration rate and weight of individual tubers; (**I**): correlation analysis between transpiration rate and yield per plant. Chl indicates total chlorophyll content; Pn indicates net photosynthetic rate; Tr indicates rate of transpiration.

Photosynthetic characteristics were significantly correlated with yield traits under different light treatments (Figure 10). Chlorophyll content was strongly positively correlated with tuber number, average tuber weight, and total yield per plant (r = 0.95, 0.94, 0.96; *p* < 0.01). The net photosynthetic rate also exhibited significant positive correlations with tuber number, average tuber weight, and total yield (r = 0.82, 0.86, 0.79; *p* < 0.01). Similarly, the transpiration rate showed strong positive correlations with tuber number, average tuber weight, and total yield (r = 0.87, 0.84, 0.81; *p* < 0.01). These findings highlight chlorophyll content, net photosynthetic rate, and transpiration rate as critical factors influencing potato yield under varying light qualities.

### 3.9. Correlations Between Stomatal Density and Yield Traits Under Different Light Qualities

**Figure 11:**
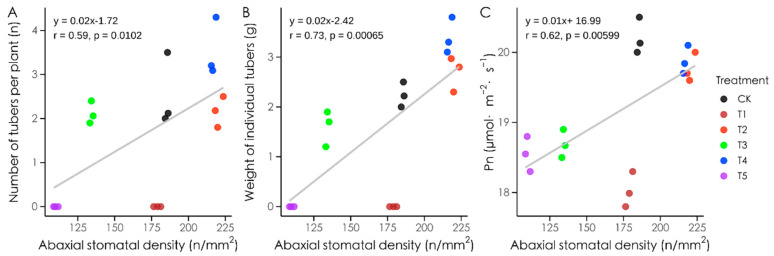
Correlation analysis of stomatal characteristics, photosynthetic rate, and yield traits in potatoes. Note: (**A**): correlation analysis between stomatal density on distal axial surface and number of potato tubers on a single plant; (**B**): correlation analysis between stomatal density on distal axial surface and weight of individual tubers; (**C**): correlation analysis between stomatal density on distal axial surface and net photosynthetic rate. Pn indicates net photosynthetic rate.

Stomatal density on the abaxial surface of Atlantic potatoes showed significant correlations with yield traits and photosynthetic rate under different light qualities (Figure 11). Stomatal density was positively correlated with the number of tubers per plant (r = 0.59, *p* < 0.05), average tuber weight (r = 0.73, *p* < 0.01), and net photosynthetic rate (r = 0.62, *p* < 0.01). These results indicate that the stomatal density on the abaxial surface influences both the photosynthetic rate and yield.

## 4. Discussion

### 4.1. Effects of Light Quality on Morphological Development of Potatoes

Light signals are perceived by photoreceptors, including phytochromes (PHY) for red and far-red light, cryptochromes (CRY) for blue and ultraviolet light, and phototropins (Phot). These photoreceptors regulate plant morphological development. Lee et al. found that the use of red light on tomatoes promoted stem elongation and increased plant height, but resulted in thin, weak stems and small, wrinkled leaves [27]. Tomohiro et al. showed that the use of blue light on lettuce thickened stems, reduced plant height, and increased leaf size and thickness [28]. While green light is often considered ineffective, some studies suggest that it promotes plant growth by enhancing photosynthesis and increasing yield [29]. Under far-red light, plants exhibited weak growth, fragile stems, and reduced root and leaf development [30]. Ultraviolet light inhibited growth by regulating gibberellin levels [31]. Our study found that red and green light increased potato plant height, but reduced stem thickness. Blue light reduced plant height, but increased stem thickness, which is consistent with previous results. Far-red and ultraviolet light treatments significantly inhibited growth, leading to stunted plants. Plants regulate their growth patterns by sensing the red/far-red light ratio (R:FR), and in low-R:FR environments, plant stems and leaves elongate to avoid shading [32]. Continuous far-red light treatment induced plants to adapt to a low-light environment by reducing stem diameter and leaf expansion [33]. The low red/far-red ratio of shade light reduces photoreceptor B activity, which allows the direct activation of transcription of growth hormone synthesis genes by the photoreceptor interaction factor (PIF), leading to a shade response. The direct interaction of PIF with DELLA proteins links gibberellin and oleuropein steroid signaling to shade avoidance [34]. Far-red light may further aggravate plant stunting by inhibiting the expression of genes related to gibberellin synthesis [35]. This is consistent with our experimental results: the growth inhibition phenomenon found here is consistent with these generalized shade avoidance effects, and continuous far-red light treatment can inhibit potato growth.

Light affects root growth by influencing leaf photosynthesis and the transport of photosynthetic products [36]. Root responses to light vary across species. Research indicates that blue light promotes root formation, as seen in tea plants [37]. Red light enhances primary and lateral root growth, increasing root length and lateral root number, although some studies report that red light inhibits root growth in barley [38]. The effects of green, far-red, and ultraviolet light on roots are unclear, but they generally inhibit root growth. In our study, red light promoted both root length and surface area in potatoes, while blue light increased root surface area without affecting root length, suggesting that blue light primarily increases root number, which is consistent with previous research. Green light inhibited root growth, reducing both root length and surface area compared to white light, in line with findings in potato tissue culture [39].

### 4.2. Effects of Light Quality on Photosynthetic Properties of Potatoes

Photosynthesis directly influences plant growth, development, and yield. The dry matter of plants primarily derives from photosynthetic products, and light is essential for its regulation. Different light qualities modulate plant photosynthetic pigment content and photosynthetic rate. Chlorophyll a and chlorophyll b are the primary pigments involved in photosynthesis. Light quality affects their formation and accumulation. Studies have shown that red and blue light increased chlorophyll content in kiwi seedlings, while green light reduced it [40]. Our study found that blue light significantly increased the chlorophyll content in potato leaves compared to white light, whereas red and green light treatments had no significant effect. Far-red and ultraviolet light treatments decreased the chlorophyll content in Atlantic potato leaves. Studies have shown that far-red light can disrupt the energy balance between PSI and PSII, leading to the accumulation of reactive oxygen species (ROS) and triggering signals for chloroplast degradation [41].

Light quality affects the photosynthetic rate through the formation of pigments and leaf development. Dezhi et al. found that cucumber plants treated with blue and red–blue light combinations had higher net photosynthetic rates than other treatments [42]. Red light inhibited photosynthetic properties in eggplant [43]. In this study, the net photosynthetic rate decreased under all light treatments compared to the control, likely due to species differences. Correlation analysis showed that chlorophyll content, net photosynthetic rate, and transpiration rate were positively correlated with tuber number, tuber weight, and plant yield.

### 4.3. Effects of Light Quality on Leaf Structure and Stomatal Characteristics of Potatoes

Different light qualities affect leaf structure. Under red light, tomato leaves become thinner, with reduced thickness of both the palisade and spongy tissues [44]. Blue light increases the thickness of both the palisade and spongy tissues in pepper leaves [45]. In our study, red and blue light treatments increased leaf thickness, with red light promoting spongy tissue thickness, which contradicts previous findings. Blue light increased the thickness of both the upper and lower epidermis. Green light did not significantly affect leaf thickness, but reduced palisade tissue thickness, while increasing spongy tissue thickness. Far-red and ultraviolet light treatments suppressed leaf growth, decreasing both leaf thickness and the thickness of palisade and spongy tissues.

Stomata regulate gas exchange between the leaf and the environment, controlling CO_2_ influx and water loss, which is essential for maintaining plant water status, leaf temperature, and photosynthesis [46]. Different light qualities influence stomatal size and density. Bernardo et al. showed that red and blue light are crucial for stomatal development [47]. Lim et al. found that combinations with blue light resulted in higher photosynthetic rates and stomatal conductance compared to red light, with both green and blue light promoting stomatal development and regulating water use efficiency [48]. In this study, blue light increased stomatal length in Atlantic potato leaves compared to white light, consistent with findings in walnut plants [49]. Far-red, red, and green light treatments did not significantly affect stomatal size compared to white light. However, studies have shown that far-red light treatment may inhibit the activity of stomatal differentiation-related transcription factors (SPCH) by interfering with the synergistic action of photoreceptors and cryptochromes in guard cells, thus leading to a decrease in stomatal density [50]. Blue light increased stomatal density, aligning with studies in other plants [51]. Our results showed that red and blue light treatments significantly increased the stomatal density in Atlantic potato leaves, consistently with previous studies. Green light inhibited stomatal development, significantly reducing stomatal density on both adaxial and abaxial surfaces compared to white light, similarly to findings in tomato leaves [52].

### 4.4. Effects of Light Quality on Yield Traits of Potato

Different light qualities regulate potato tuber formation in various ways. Red light delays tuber formation, while blue light advances it by 2–5 days [53]. Blue light treatment significantly increases the tuber number and weight per plant [54]. Red light inhibits tuber morphology and enlargement, with short exposure being reversible by far-red light, but long exposure being irreversible [55]. Rahman et al. found that green light treatment resulted in a higher number of tubers and greater tuber weight compared to white, red, and blue light treatments [54]. Our contrasting results may be attributed to differences in variety; however, a more likely explanation lies in the differences in treatment intensity and duration. Additionally, numerous studies have demonstrated a dose-dependent dual effect of light quality, which needs to be further verified through targeted experiments. Red light reduced the tuber number, but the weight of individual tubers and the yield per plant were not significantly different from those observed for white light, indicating that red light does not affect yield by reducing the number of tubers. Far-red and ultraviolet light treatments prevented tuber formation. Studies have shown that far-red light inhibits the expression of StSP6A, a key gene in tuber formation, by activating the phyA signaling pathway, thereby blocking photoperiodic tuber initiation [56,57]. Green light inhibited tuber number and yield. Green light can penetrate deeper into the plant canopy compared to other light spectra. In lower-positioned leaves that receive less red and blue light due to self-shading, green light can enhance photosynthetic efficiency [58]. Although green light can enhance photosynthesis in lower leaves, excessive green light exposure may disrupt the overall balance of plant growth and development [59]. For example, it may affect the source–sink relationship in the plant. An over-emphasis on photosynthesis in lower leaves due to green light may lead to an imbalance in the distribution of photosynthates. The excess photosynthates produced in the lower leaves may not be efficiently transported to the developing tubers (the main yield-determining organs in potatoes), resulting in reduced productivity. Blue light promoted both tuber number and weight. Our previous study also found that blue light significantly increased the tuber number, average tuber weight, and harvest index in potato microtubers [60].

### 4.5. Correlations Between Photosynthetic Characteristics and Yield Traits Under Different Light Qualities

Chlorophyll is the primary pigment for light-harvesting in photosynthesis. Chlorophyll-rich plants can capture more light energy, which is then converted into chemical energy (ATP and NADPH) during light-dependent reactions [61]. These energy-rich molecules are essential for the Calvin–Benson cycle (light-independent reactions), where carbon dioxide is fixed to form carbohydrates. A higher chlorophyll content generally leads to more efficient photosynthesis, and thus higher yields. We found that chlorophyll content was strongly positively correlated with tuber number, average tuber weight, and total yield per plant. Our results are consistent with the literature.

Stomatal conductance is crucial as it controls the entry of carbon dioxide into the leaf, which is the substrate for photosynthesis. Higher stomatal conductance allows more carbon dioxide to enter the leaf, promoting the rate of photosynthesis [62]. At the same time, it is also related to transpiration. Transpiration creates a transpiration pull, which helps in the uptake of water and nutrients from the soil. Adequate water and nutrient supply is vital for plant growth and photosynthesis, and all these factors ultimately contribute to higher yields. We discovered that the net photosynthetic rate also exhibited significant positive correlations with tuber number, average tuber weight, and total yield.

Transpiration promotes the absorption and transportation of water and nutrients. The transpiration pull helps the roots to absorb water and nutrients and maintain the water balance within the plant, providing a basis for plant growth and the accumulation of photosynthetic products [63], as well as regulating the leaf temperature. Through the evaporation of water, transpiration prevents the leaves from being burned, and maintains the activity of photosynthetic enzymes and the stability of the photosynthetic apparatus, ensuring the efficient progress of photosynthesis and thus increasing the yield. In addition, it affects stomatal movement. It interacts with stomatal conductance to balance photosynthesis and water use, which has different impacts on the yield under different water conditions. Lastly, it participates in plant hormone regulation, influencing the transportation, distribution, and signal transduction of hormones, regulating plant growth, development, and adaptation to stress, and thus affecting the yield. We found that the transpiration rate showed strong positive correlations with tuber number, average tuber weight, and total yield.

### 4.6. Future Research Directions

In the future, on the basis of this study, molecular biology technology can be used to further explore the light signal transduction pathway, the expression mechanism of genes related to light activation, and the functions of light regulatory factors, including how light signals activate gene expression through receptors such as cryptochrome and phytochrome, and regulate chloroplast pigment synthesis and photosynthetic enzyme activity. In addition, the regulatory effects of light on endogenous hormones can be studied to reveal their molecular mechanisms in plant growth and yield. At the same time, high-throughput sequencing and gene editing technologies can be combined to analyze the regulatory effects of blue and red light on the expression of key genes, and how these changes affect the physiological and metabolic processes of plants. These studies will provide theoretical support at the molecular level for optimizing light conditions and increasing potato yield.

## 5. Conclusions

The results of this study showed that different light qualities had significant effects on the growth, photosynthetic characteristics, and yield traits of potatoes, with blue and red light exhibiting prominent promoting effects under experimental conditions. Blue light enhances transpiration by expanding the stomatal size and increasing the stomatal density. It also significantly increases the chlorophyll content in the leaves and enhances the thickness of both the upper and lower epidermis, contributing positively to the improvement of photosynthetic structures. Additionally, blue light significantly improves the root surface area and the number of tubers per plant, promoting tuber growth; the weight of individual tubers and the overall yield per plant were notably higher under the blue light treatment, compared to the other light treatments. Red light demonstrates clear advantages in promoting plant height and stem thickness. By increasing the thickness and enhancing the tight arrangement of the spongy tissue in leaves, it markedly enhances the growth vigor of aboveground parts. Moreover, red light does not significantly increase the weight of individual tubers. Although the number of tubers per plant decreases slightly, the overall yield potential remains substantial. In contrast, green light, while aiding chlorophyll synthesis and plant height growth, has relatively weak effects on potato growth and significantly inhibits yield. Far-red (740 nm) and ultraviolet light (390 nm), on the other hand, exhibit inhibitory effects on both growth and yield. In conclusion, red and blue light are key light qualities for promoting potato growth and enhancing yield. The findings of this study provide a theoretical foundation for further research on the mechanisms of light-regulated potato growth, light condition optimization for enhanced productivity, and modern agricultural light management strategies.

## Figures and Tables

**Table 1:** Different light quality settings.

Treatment Number	CK	T1	T2	T3	T4	T5
Different light qualities	White light	Far-red light	Red light	Green light	Blue light	Ultraviolet light
Wavelengths (nm)	400–760	740	660	520	450	390
FWHM (nm)	21–150	100	24	30	26	30

Note: The wavelengths of the treatments were measured as follows: 400–760 nm for the CK, 740 nm for the far-red light, 660 nm for the red light, 520 nm for the green light, 450 nm for the blue light, 390 nm for the ultraviolet light. FWHM: Full Width at Half Maximum.

**Table 2:** The effects of different light qualities on the photosynthetic characteristics of potato leaves.

Variety	Treatment	Chl (mg/g)	Pn (μmol·m^−2^·s^−1^)	Tr (mmol·m^−2^·s^−1^)
Atlantic	CK	1.72 ± 0.01 b	20.21 ± 0.22 a	1.62 ± 0.02 bc
	T1	1.31 ± 0.04 c	18.03 ± 0.40 d	0.96 ± 0.05 d
	T2	1.72 ± 0.05 b	19.77 ± 0.16 b	1.82 ± 0.04 ab
	T3	1.74 ± 0.04 b	18.69 ± 0.02 c	1.51 ± 0.05 c
	T4	2.07 ± 0.05 a	19.88 ± 0.02 ab	1.97 ± 0.25 a
	T5	1.36 ± 0.01 c	18.55 ± 0.16 c	1.47 ± 0.06 c

Note: Chl indicates total chlorophyll content; Pn indicates net photosynthetic rate; Tr indicates rate of transpiration. CK: white light treatment; T1: far-red light treatment; T2: red light treatment; T3: green light treatment; T4: blue light treatment; T5: ultraviolet light treatment. The data in the table are displayed as the average ± standard deviation; different lowercase letters indicate significant differences at *p* < 0.05.

**Table 3:** The effects of different light qualities on the stomatal size of potato.

Variety	Treatment	Adaxial Stomatal Length (μm)	Adaxial Stomatal Width (μm)	Abaxial Stomatal Length (μm)	Abaxial Stomatal Width (μm)
Atlantic	CK	36.11 ± 1.94 ab	20.35 ± 2.55 ab	25.89 ± 2.67 bc	16.15 ± 1.80 b
	T1	35.75 ± 3.13 b	22.53 ± 1.50 ab	28.74 ± 3.61 ab	17.28 ± 1.76 ab
	T2	34.10 ± 3.62 b	21.99 ± 2.16 ab	24.73 ± 3.00 bc	16.36 ± 2.32 ab
	T3	34.45 ± 3.47 b	24.57 ± 2.99 a	22.42 ± 2.11 c	17.30 ± 2.43 ab
	T4	42.09 ± 3.50 a	23.74 ± 2.81 a	33.13 ± 2.47 a	20.92 ± 2.43 a
	T5	30.12 ± 2.01 b	18.50 ± 1.25 b	25.23 ± 3.00 bc	15.48 ± 2.39 b

Note: CK: white light treatment; T1: far-red light treatment; T2: red light treatment; T3: green light treatment; T4: blue light treatment; T5: ultraviolet light treatment. The data in the table are displayed as the average ± standard deviation; different lowercase letters indicate significant differences at *p* < 0.05.

**Table 4:** The effects of different light qualities on the yield components of potato plants.

Variety	Treatment	Number of Tubers per Plant (n)	Weight of Individual Tubers (g)	Yield per Plant (g)
Atlantic	CK	2.54 ± 0.12 b	2.24 ± 0.07 b	5.69 ± 0.09 b
	T1	——	——	——
	T2	2.16 ± 0.14 c	2.69 ± 0.12 b	5.80 ± 0.20 b
	T3	2.12 ± 0.26 c	1.6 ± 0.24 c	3.42 ± 0.76 c
	T4	3.53 ± 0.12 a	3.40 ± 0.53 a	11.97 ± 1.72 a
	T5	——	——	——

Note: “——” means no potatoes. CK: white light treatment; T1: far-red light treatment; T2: red light treatment; T3: green light treatment; T4: blue light treatment; T5: ultraviolet light treatment. The data in the table are displayed as the average ± standard deviation; different lowercase letters indicate significant differences at *p* < 0.05.

## Data Availability

The original data presented in the study are included in the article; further inquiries can be directed to the corresponding author.
